# Ontogeny of the facial phenotypic variability in Mexican patients with 22q11.2 deletion syndrome

**DOI:** 10.1186/s13005-019-0213-9

**Published:** 2019-12-11

**Authors:** Arodi Farrera, María Villanueva, Alfredo Vizcaíno, Patricia Medina-Bravo, Norma Balderrábano-Saucedo, Mariana Rives, David Cruz, Elizabeth Hernández-Carbajal, Javier Granados-Riveron, Rocío Sánchez-Urbina

**Affiliations:** 1 Instituto de Investigaciones Antropológicas, UNAM, Ciudad de México, Mexico; 2 Departamento de Cardiología, Hospital Infantil de México Federico Gómez, Ciudad de México, Mexico; 3 Departamento de Endocrinología, Hospital Infantil de México Federico Gómez, Ciudad de México, Mexico; 4 Laboratorio de Investigación en Cardiopatías Congénitas, Hospital Infantil de México Federico Gómez, Ciudad de México, Mexico; 5 Laboratorio de Genómica, Instituto Nacional de Cardiología Dr. Ignacio Chávez, Ciudad de México, Mexico; 6 Escuela Superior de Medicina del Instituto Politécnico Nacional, Ciudad de México, Mexico; 7 Laboratorio de Investigación en Genómica, Genética y Bioinformática. Hospital Infantil de México Federico Gómez, Ciudad de México, Mexico; 8 Laboratorio de Investigación en Biología del Desarrollo y Teratogénesis Experimental, Hospital Infantil de México Federico Gómez, Dr. Márquez No.162 Col. Doctores, C.P.:06720 Delegación Cuauhtémoc, DF Mexico

**Keywords:** 22q11.2, Ontogeny, Facial features, Allometry

## Abstract

**Background:**

22q11.2 deletion syndrome is a medical condition that results from genomic loss at chromosome 22. Affected patients exhibit large variability that ranges from a severe condition to mild symptoms. In addition, the spectrum of clinical features differs among populations and even within family members. The facial features related to this syndrome are not an exception, and although part of its variation arises through development, few studies address this topic in order to understand the intra and inter-population heterogeneities. Here, we analyze the ontogenetic dynamics of facial morphology of Mexican patients with del22q11.2 syndrome.

**Methods:**

Frontal facial photographs of 37 patients (mean age = 7.65 ± 4.21 SE) with del22q11.2DS and 200 control subjects (mean age = 7.69 ± 4.26 SE) were analyzed using geometric morphometric methods. Overall mean shape and size differences between patients and controls were analyzed, as well as differences in ontogenetic trajectories (i.e. development, growth, and allometry).

**Results:**

We found that Mexican patients show typical traits that have been reported for the Caucasian population. Additionally, there were significant differences between groups in the facial shape and size when all the ontogenetic stages were considered together and, along ontogeny. The developmental and allometric trajectories of patients and controls were similar, but they differed in allometric scaling. Finally, patients and controls showed different growth trajectories.

**Conclusion:**

The results suggest that the typical face of patients with del22q11.2DS is established prenatally; nonetheless, the postnatal ontogeny could influence the dysmorphology and its variability through size-related changes.

## Background

The human head is considered a complex morphological structure because it results from the interaction of genetic and environmental factors, and its phenotypic variation is structured through ontogeny by the coordinated development of the different traits that compose it. Disruptions of this highly coordinated ontogenetic processes or the introduction of new interactions could lead to dysmorphogenesis [1]; for instance, chromosomal abnormalities such as deletions that cause abnormal ontogeny and severe congenital anomalies. Velocardiofacial syndrome (VCFS), also known as 22q11.2 deletion syndrome (22q11.2DS), is an example of a group of defects caused by the deletion of a small segment of chromosome 22, with a frequency estimated to be between 1/2000 and 1/4000 of live newborns, and with above 90% of cases being the result of de novo mutations during early fetal development and some of them inherited following an autosomal dominant pattern [2, 3].

22q11.2DS presents great clinical variability [4], as has been described in familial studies [2, 5, 6], and is most frequently associated with congenital heart disease (CHD), which is present in 75–85% of patients [2, 7]. The most common clinical characteristics of this syndrome, in addition to CHD, are hypoplasia or aplasia of the thymus, neonatal hypocalcemia, immunological disorders, velopharyngeal insufficiency, palate abnormalities, learning and behavioral disorders, and abnormal facial features [2–4, 6, 8–12].

The diagnosis of patients with 22q11.2DS is suspected in individuals with cardiac defects or vertebral anomalies, which is confirmed if the deletion is identified by using FISH (Hibrdation in situ Fluorescent), MLPA (Multiplex Ligation-dependent probe Amplification), aCGH (array Comparative Genomic Hybridization) or GWAS (Whole Genome Association Study) (www.orphanet.net.), in addition, it can be made according to the aforementioned phenotypic characteristics and, with the support of the facial phenotype, differential diagnosis can be accomplished. The “typical” facial features of this syndrome include malar hypoplasia, “puffy” eyelids, abnormalities of the external ear, bulbous nasal tip, prominent nasal root, hypoplasia of the nasal wings, hypertelorism, upward oblique palpebral fissures and small mouth [3, 11, 13–16].

However, one of the difficulties associated with the use of facial appearance for the diagnosis of this syndrome is the significant variation observed across patients. For instance, the degree of severity of the typical facial appearance of patients with 22q11.2DS may differ among populations [13].

African-Americans and other patients of African descent show a less pronounced phenotype [17–20] compared with Caucasian patients; in contrast, the characteristic face of Chinese patients is strongly associated with 22q11.2DS and forms an indicative factor for direct diagnosis [21]. Similar variability has been reported between parents and offspring [2, 5, 22] or even between monozygotic twins [23, 24]. Moreover, changes in facial appearance with increasing age have been demonstrated in these patients, with more subtle manifestations observed in adults than in children [25–27]. These findings are relevant, since they suggest that, in addition to the early contribution of genetic factors to establish the typical facial features of different syndromes [28], postnatal ontogeny could have an important influence on facial morphological variation. In the non-affected population, some amount of facial variation arises by different mechanisms along ontogeny (i.e. development, growth, and allometry) through variations in the rate and timing of shape and size changes [29]. In this way, ontogeny is often seen as a path defined by the relationship among these factors, in which development and growth processes depict, respectively, trajectories of change in shape and size through time. In addition, allometry implicitly refers to the former mechanisms given that it is the effect that variation in the size of an organism has on its shape variability.

The facial structure is the last to mature during postnatal ontogeny, so, its variability would be expected to be more influenced by differences in those ontogenetic mechanisms. Some studies have confirmed the importance of postnatal ontogeny in facial variation of contemporary populations [30, 31]. For example, Vioarsdóttir *et al*. reported that differences among populations are generated by divergence of such ontogenetic trajectories or, in the case of populations with similar trajectories [30], from differences in ontogenetic scaling, i.e., morphological differences, because the adult phenotypes of one population occupy a different space with respect to that occupied by other populations within the same ontogenetic trajectory. In addition, Freidline *et al*. found that there are more evident differences in the rates of growth and development between populations during early ontogeny than in later stages [31].

In this context, the degree of facial dysmorphology of 22q11.2DS individuals could be affected by the disruption of the ontogenetic patterns present in the general population, as has been shown for the facial morphology that is characteristic of Williams syndrome, given that the facial growth of patients is slow compared with that of non-affected individuals [32]. In contrast, individuals with Sotos syndrome show morphological growth-related changes similar to those of the general population; thus, the typical facial features established during early ontogeny remain consistent over time [33]. Therefore, it is likely that there may be specific ontogenetic patterns for each syndrome. The analysis of the manner by which the adult morphology is reached through ontogeny could aid in the diagnosis of affected individuals and the understanding of the causes of the facial morphological features associated with 22q11.2DS and their inter- and intra-population heterogeneities.

Among the clinical characteristics already mentioned, growth deficiency is commonly observed in patients [34, 35]. This deficiency has been suggested to be a result of other existent characteristics (e.g. feeding difficulties) in the syndrome, and genetic factors related to the deletion [34, 36, 37]. In both cases, this growth impairment would disturb the ontogenetic trajectory of patients and therefore could be considered as a factor influencing the facial variability of this syndrome.

The objective of this study is to quantitatively explore the facial morphology and its variation in a sample of Mexican children and adolescents with 22q11.2DS, and to examine how this variation changes along ontogeny. In order to do so, we compared, by means of geometric morphometrics, the facial morphology of a group of patients with that of control subjects, emphasizing the influence of the ontogenetic process in the establishment of the dysmorphic face.

## Patients and methods

### Participants

The patient group included individuals without palatal abnormalities or cleft lip and palate surgery to avoid increasing facial variability due to the presence of this disorder. This group included 37 children (eighteen girls and nineteen boys) between the ages of 2 and 17 years of both genders and from Mexico City and the surrounding states (State of Mexico, Hidalgo, and Queretaro) with clinical diagnoses of VCFS and 22q11.2DS, as established by fluorescent in situ hybridization (FISH) using the Tuple1 probe (Vysis®). Patients with CHD were recorded, as well as their type of CHD. The patients were compared with a control group that included 200 age-matched individuals mainly from Mexico City and the State of Mexico. In order to explore the morphological differences through ontogeny, both groups were subdivided into different age groups that roughly reflect the major stages of development (i.e. early childhood, middle childhood, and adolescence), and, as far as possible, with balanced sample size (Table 1). This study was reviewed and approved by the local ethics committees. Informed consent was obtained from both groups.

**Table 1:** Description of the sample according to age and sex of the individuals

		Patients		Controls	
Group	Age range	Female	Male	Female	Male
G1	< 5 years	8	4	24	17
G2	5 to 10.9 years	8	9	62	52
G3	11 to 20 years	2	6	21	24
	Total	18	19	107	93

### Measurement acquisition

Frontal facial photographs were taken of the individuals with a standardized protocol that included the placement of the faces according to the Frankfort plane and image capture using a Sony® Alpha 33 digital camera with a Sony® 50 mm lens placed at a constant distance of 1.5 m from the face, with a scale placed at ear height.

**Fig. 1:**
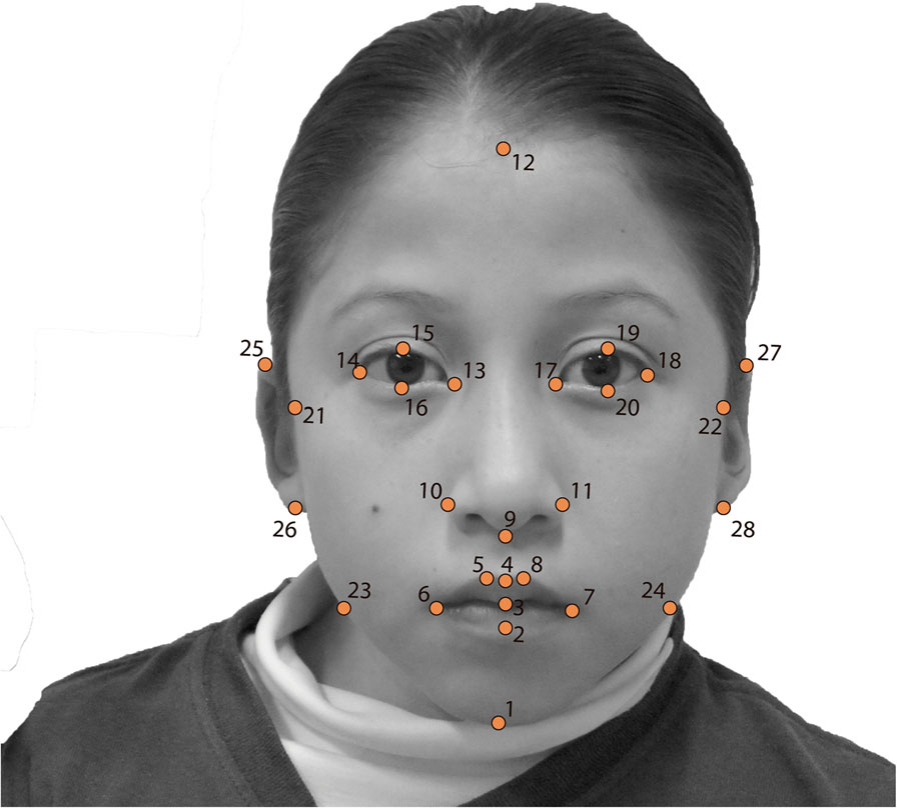
Landmarks used in this study (see definitions in Table 2)

Using the program TPSDIG v.2.16, 28 landmarks were placed, including six over the sagittal line and 22 bilateral landmarks [37] (Table 2, Fig. 1). The resulting coordinates were analyzed using geometric morphometric (GM) and multivariate analyses. GM analysis is a technique used for the capture, analysis, and visualization of shape change, with advantages over traditional morphometric techniques [38]. Shape information is extracted by Procrustes superimposition, which removes variations in location, scale and rotational effects from coordinates in 2D or 3D but retains size information (i.e., centroid size) for use in subsequent analyses. Centroid size is defined as the square root of the summed squared distances of all landmarks from their centroid; it is used as the scale of the landmark configuration [39]. Subsequent to this adjustment, the standardized coordinates are analyzed with multivariate statistical methods. During the analysis, the geometric relationships among variables are preserved, thereby facilitating the visualization of shape change. Together, these features make GM analysis a powerful technique for the assessment of morphological variation.

**Table 2:** Soft tissue facial landmarks used in the study

No	Landmarks	Definition
Sagittal Line		
1	Gnation	The lowest point in the midline on the lower border of the chin.
2	Labiale inferius	The midpoint of the vermilion border of the lower lip.
3	Stomion	The midpoint of the labial fissure when the lips are closed naturally.
4	Labiale superius	The midpoint of the vermilion border of the upper lip.
9	Subnasale	The junction between the lower borders of the nasal septum.
12	Trichion	Midpoint of the hairline.
Bilateral		
5,8	Crista Philtre	The point of the crest of the philtrum, above the vermilion border.
6,7	Chelion	The outer corner of the mouth where the upper and lower lips meet.
10,11	Alare crest	The most lateral point on the nasal ala.
13,17	Endocanthion	The inner corner of the eye fissure where the eyelids meet.
14,18	Exocanthion	The outer corner of the eye fissure where the eyelids meet.
15,19	Palpebral superius	The highest point on the upper margin of the middle portion of the eyelid.
16,20	Palpebral inferius	The lowest point on the upper margin of the middle portion of the eyelid.
21,22	Photographic zygion	The most external point on the margin of the face below the eyes.
23,24	Photographic gonion	The most outward projecting point on the face along the horizontal axis of the mouth.
25,27	Superaurale	The highest point on the upper edge of the helix of the ear.
26,28	Subaurale	The lowest point on the lobe of the ear.

The analysis was performed by taking into consideration the bilateral symmetry exhibited by the face, i.e. Procrustes superimposition was performed with the original and mirrored configurations of each set of landmarks, allowing the morphological variation related with asymmetry to be ruled out, as it is of no interest in this study. In this manner, a totally symmetrical morphology was analyzed [40].

### Statistical analysis

First, we carried out a principal component analysis (PCA) to visualize the main axis of facial variability within our sample. The analysis generates a new set of variables called principal components (PCs) that are functions of the covariances among the original variables and that account for most of the sample variation. We considered only those PCs that explained above 10% of the total variance.

In order to explore the contribution of development (shape change with age) to the facial variation within our sample, we used a two-way multivariate analysis of variance (two-way MANOVA) to compare facial shape differences using the patient-control status and the age group (i.e. G1, G2, G3) as factors. Moreover, the developmental trajectory was analyzed in detail as multivariate trajectory in phenotype space. In this analysis, the vector that connects the phenotypic means between age groups is determined for patients and controls, and its attributes (i.e. magnitude of phenotypic vector, vector direction, vector shape) are statistically examined pairwise by permutation test with 1000 random permutations [41]. Furthermore, we explored the relative change differences through ontogeny, by computing the Euclidean distance between the mean shapes of each age group obtained in the latter analysis. Finally, we used Goodall’s F-test to evaluate mean shape differences between patients and controls at each age group, using a resampling procedure with 400 random permutations to assess its significance.

To assess the contribution of the growth process (size change with age), we performed a two-way analysis of covariance (two-way ANCOVA) with facial size (i.e. centroid size in cm) as the dependent variable, and the patient-control status and age as factors. The significance of the null model was assessed using 400 random permutations. In addition, the growth trajectory between groups was analyzed by linear regression of the log centroid size (log CS) against individual age (as continuous variable). Finally, we compared the relative scaling in facial size between groups as percentage, with the estimate of the ratio of patients’ size at a given age relative to controls in the same stage.

Allometry (shape change with size) was assessed using multivariate analysis of covariance (MANCOVA) of the facial shape, using log CS and patient-control status as factors. The statistical significance was evaluated using 400 random permutations. Finally, the allometric trajectory was analyzed by a multivariate regression of shape variables against log CS and of predicted values against log CS. The regression score can be interpreted as the shape variable that is most strongly associated with log CS [42], while the predicted shape represents the first principal component of the predicted values calculated from the multivariate regression model [43]. All morphometric and statistical analyses were performed using the R package [44], ‘Geomorph’ version 3.0.3 [45] .

## Results

The frequency of CHD in the patients was 91.9%, with tetralogy of Fallot (37.8%) and truncus arteriosus (19%) being the most common conditions. In addition, stenosis of the pulmonary artery (8.1%), interruption of the aortic arch type B (5.4%), interventricular communication (5.4%), aortic valve stenosis (2.7%), interatrial communication plus interventricular communication (2.7%), and patent ductus arteriosus (2.7%) were observed. Only a small number of patients did not have CHD (8.1%).

**Fig. 2:**
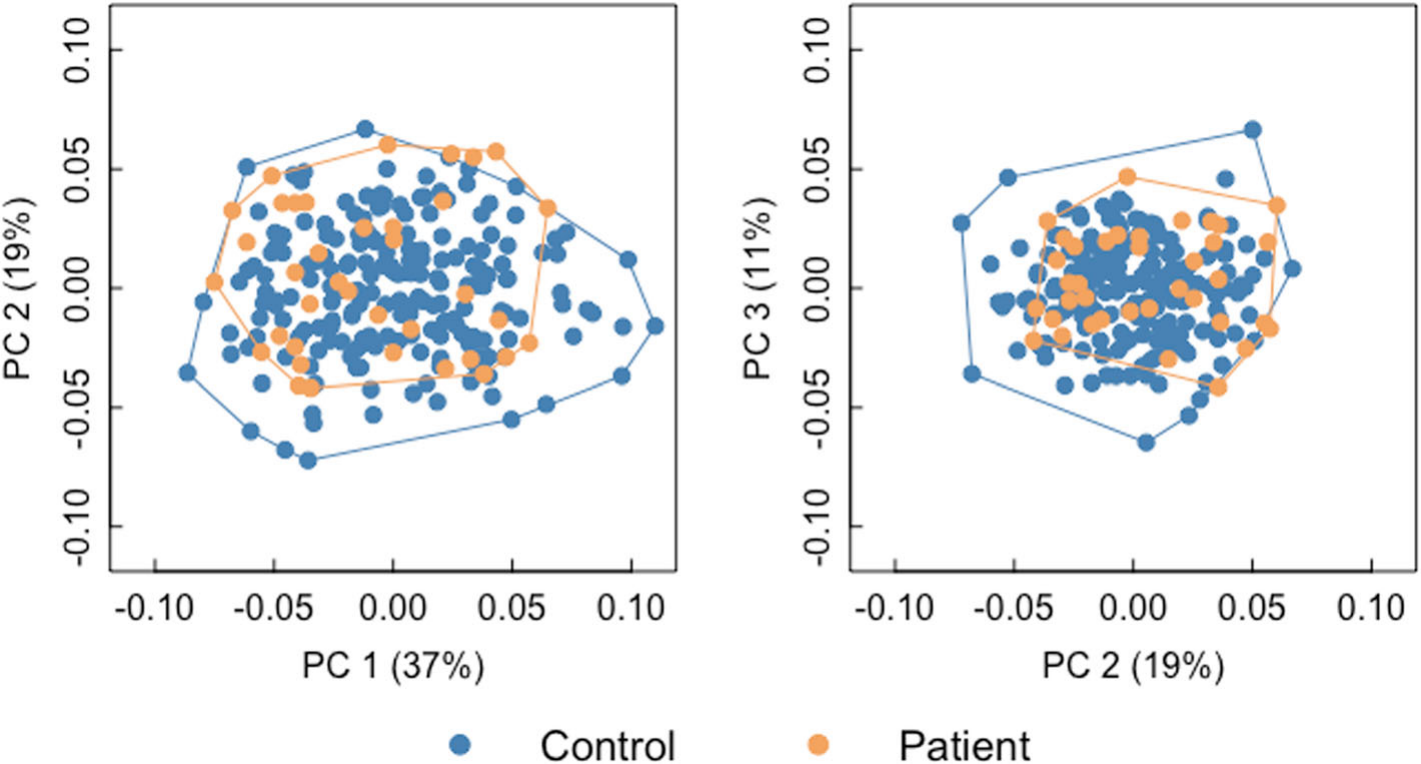
Scatterplot of first three principal components of a PCA. Convex hulls are drawn for patients (orange) and controls (blue)

The first three PCs represent 66.9% of the accumulated variation. Visual inspection of the convex hull polygons (Fig. 2) shows that facial covariation patterns of patients lie within that of controls. Moreover, the differences in the facial covariance structure between groups are not the main axis of variation (i.e. PC1, *F* (1, 235) = 2.51, *p* = 0.09; PC2, *F* (1, 235) = 1.46, *p* = 0.24; PC3, *F* (1, 235) = 0.99, *p* = 0.31) in the sample.

The two-way MANOVA showed that there was statistically significant difference in shape variables between patients and controls (*F* (1, 233) = 9.15, *p* = 0.001) and among age groups (*F* (1, 233) = 27.42, *p* = 0.001), while the interaction between both effects was not significant (*p* = 0.289). The latter result was confirmed with the developmental trajectory analysis, which showed no differences between groups, neither in the magnitude (distance = 0.0077, *p* = 0.654), the direction (angle = 30°, *p* = 0.233) or the shape (shape = 0.1882, *p* = 0.435) of the developmental vector. These results mean that, overall, both groups have a similar development with facial differences among age groups (see below), and that average facial features are different between patients and controls.

**Fig. 3:**
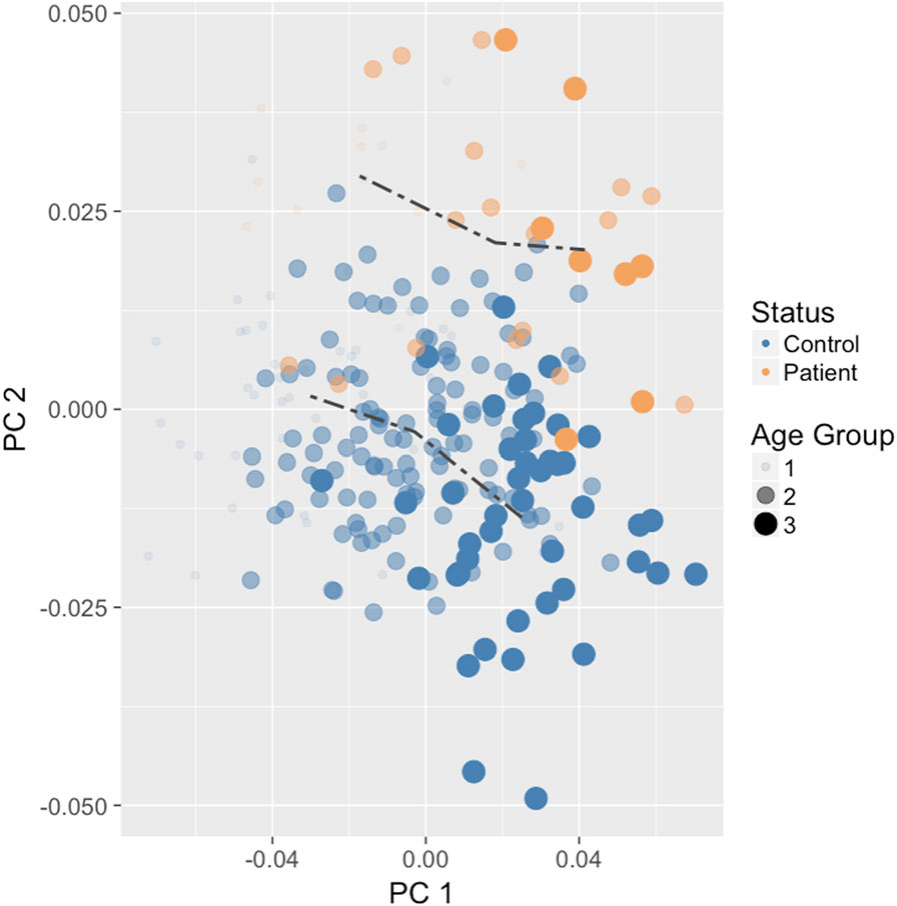
Developmental trajectory for patients and control groups projected onto two principal components of shape for patients and controls

**Fig. 4:**
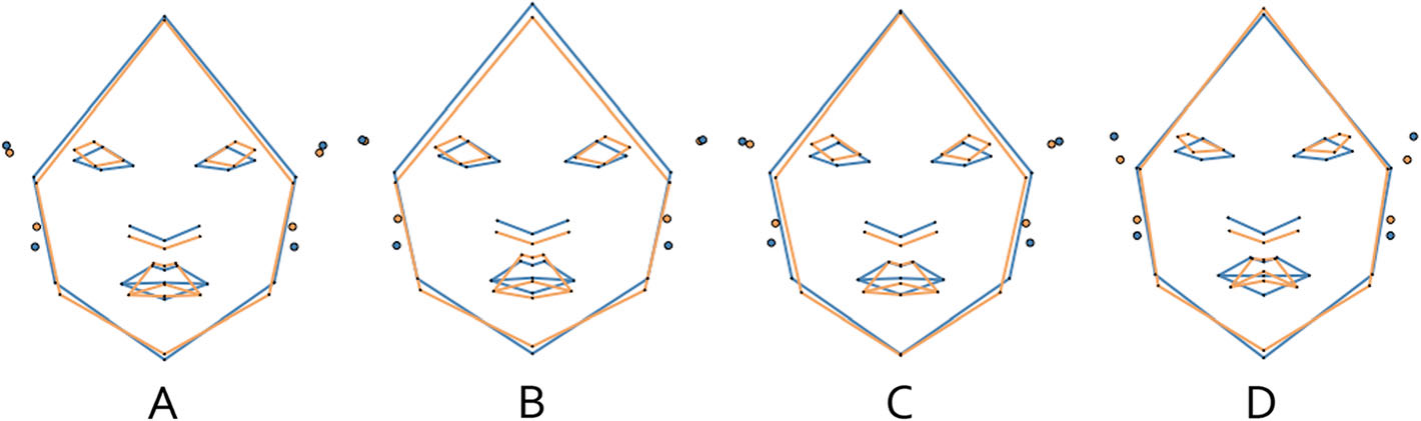
Wireframe showing the differences in mean morphological configurations between patients (orange) and controls (blue) when **a** all the age groups were considered together and at **b** G1, **c** G2, and **d** G3

However, visual inspection of relative magnitude of shape change exhibited within groups during ontogeny shows slightly different patterns (Fig. 3). There is a greater amount of shape change between G1 and G2 in patients (Euclidean distance = 0.0364) than in controls (Euclidean distance = 0.0276), and a smaller amount of shape change between G2 and G3 in patients (Euclidean distance = 0.0235) than in controls (Euclidean distance = 0.0302). In addition, when we compared mean shapes between groups within each age group, we found statistically significant differences at G1 (Goodall’s *F* = 2.73, *p* = 0.044), G2 (Goodall’s *F* = 4.70, *p* = 0.002) and G3 (Goodall’s *F* = 3.53, *p* = 0.009). In other words, patients and controls differ in facial shape at each ontogenetic stage (Fig. 4, Table 3). In general, patients have a slight hypertelorism, upslanting palpebral fissures, downward labial commissures, smaller ears, reduced philtrum, and a longer midface than individuals in control group. However, the upslanting palpebral fissures, the hypertelorism and the longer midface are more evident from G2 onwards (Fig. 4c), while the low-set position of the ears and a narrower nasal ala are observed only at G3 (Fig. 4d).

**Table 3:** Clinical features found among the age groups of patients compared to the control group

Clinical characteristics	All patients	G1	G2	G3
Downward labial commissures	+	+		
Hypertelorism	+		+	
Longer midface	+		+	
Low-set position of the ears	+			+
Narrower nasal ala	+			+
Reduced philtrum	+	+		
Smaller ears	+	+		
Upslanting palpebral fissures	+		+	

The + indicates from which age onwards each trait is observed, *p < 0.005*

**Fig. 5:**
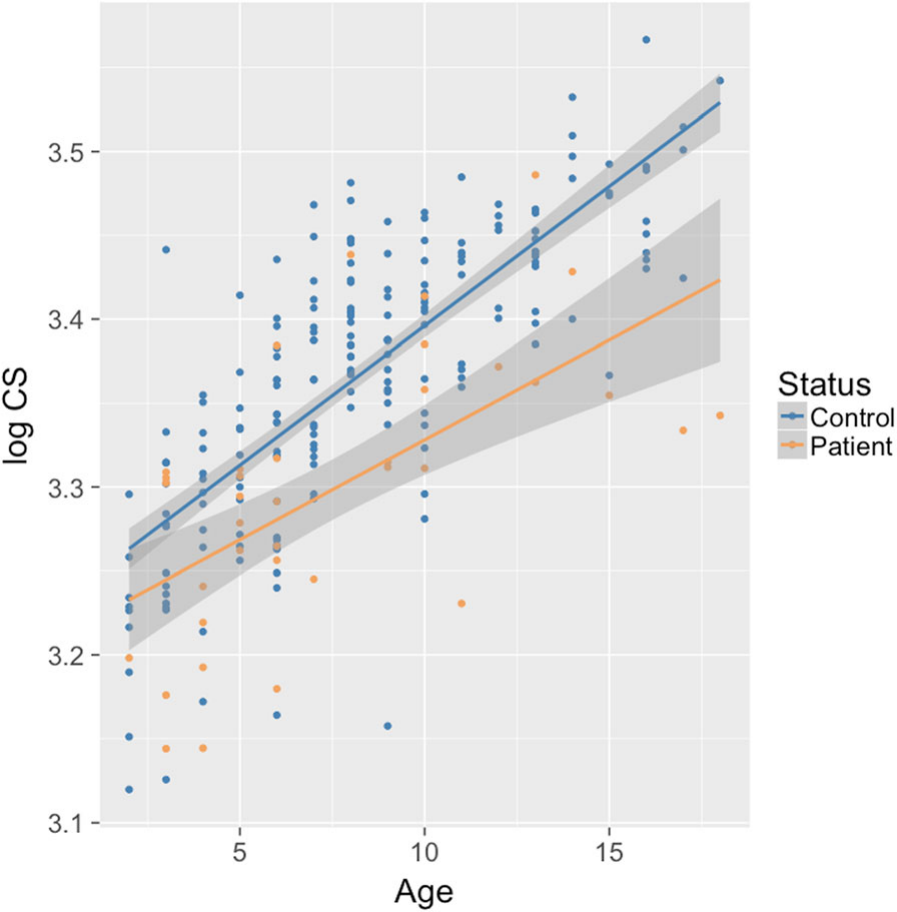
Growth trajectory for patients and control groups. Log centroid size as a function of age

The two-way ANCOVA showed statistically significant interaction effect between the patient-control status and age on facial size (F (1, 233) = 5.24, *p* = 0.05), with an overall fit of the model of R^2^ = 0.53. Figure 5 shows the scatterplot of log centroid size against age with a regression line fitted for each group (patient’s R^2^ = 0.38; control’s R^2^ = 0. 59). The percentage of the mean facial size of patients at G1 relative to that of controls at the same age stage was 96.4%, while at G2 was 94.9%, and at G3 was 91.4%. Therefore, there are differences in facial size between patients and controls that increase as the age progresses.

**Fig. 6:**
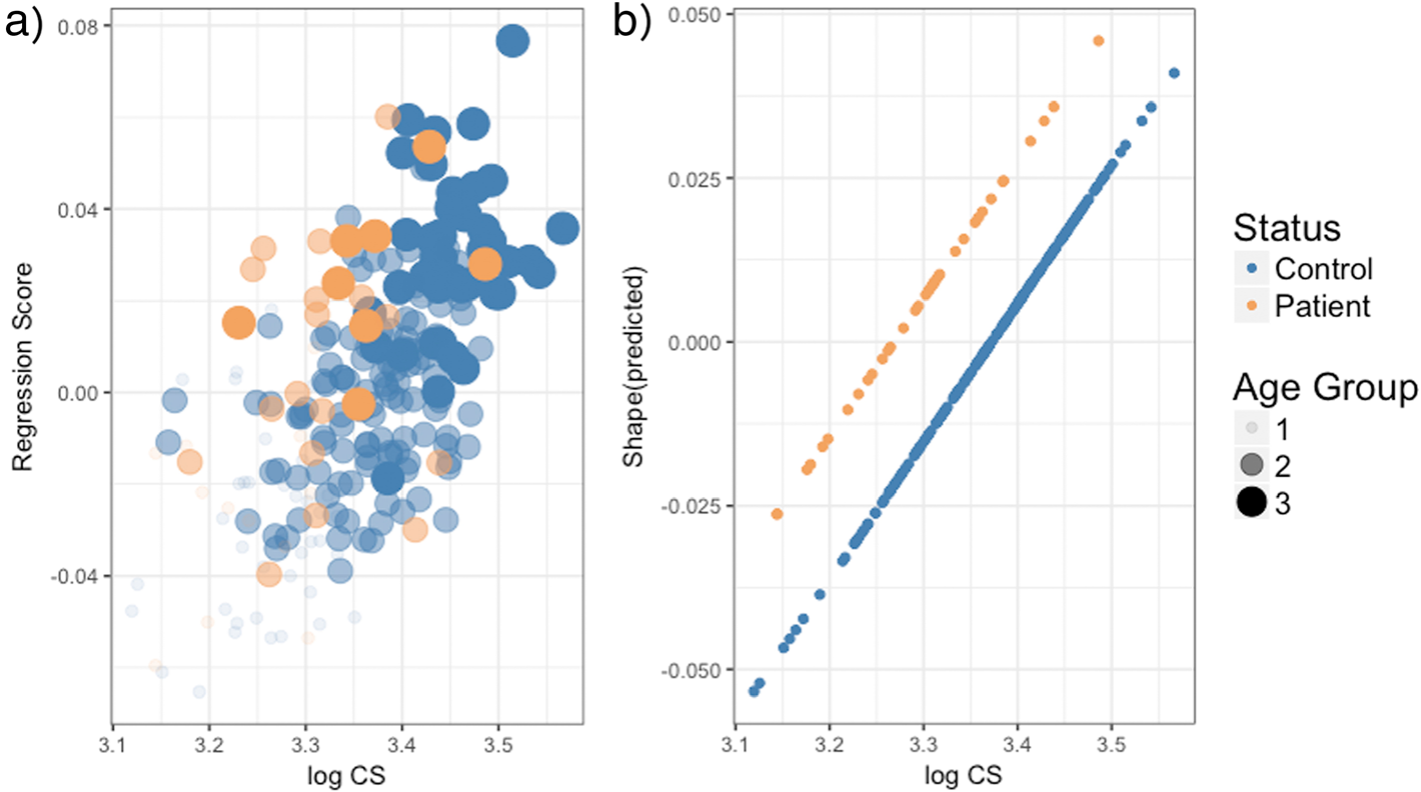
Allometric trajectory for patients and control groups. **a** Regression scores of facial shape as a function of log CS, **b** predicted values of the facial shape versus log CS

The MANCOVA that assessed the allometric contribution of shape variation showed statistically significant effect of facial size (*F* (1,233) = 19.03, *p* = 0.01, R^2^ = 0.07) and patient-control status (*F* (1,233) = 10.59, *p* = 0.01, R^2^ = 0.04), but no significant interaction (*p* = 0.23) between these effects. To facilitate comparison, Fig. 6 shows a) the scatterplot between regression scores against log facial size and B) the predicted values for patients and controls separately.

**Fig. 7:**
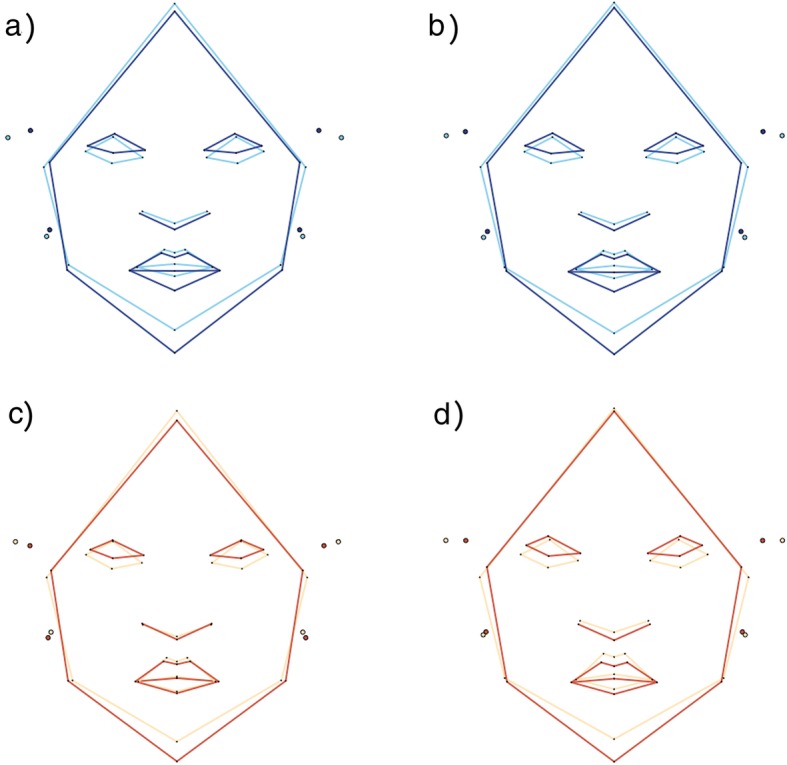
Facial shape changes related to development in (**a**) controls and (**c**) patients; and changes related to allometry in (**b**) controls and (**d**) patients. The light color depicts the youngest individuals and the dark color the oldest

Finally, facial shape differences due to development and allometry are shown in Fig. 7. In controls, facial shape influenced by development (Fig. 7a) is related to elongated faces, especially due to changes in the lower third. The thickness of the lips, the length of the philtrum and the protrusion of ears change along development as well. Similar changes are observed when allometry (Fig. 7b) is analyzed. By contrast, facial shape changes differ in patients whether development (Fig. 7c) or allometry (Fig. 7d) is analyzed. In the first case, patients show lengthening of the facial traits that is similar to that observed in controls (i.e. in the middle and lower third) but this change is only related to the facial outline. That is, neither the length of the philtrum, nor the thickness of the lips are greatly modified, which contrasts with what is observed of facial changes due to allometry. Allometric changes in patients show similar lengthening of the facial outline but changes in lips and philtrum are present.

## Discussion

We conducted a quantitative analysis of the ontogenetic dynamics of the facial morphology of Mexican children and adolescents with 22q11.2DS compared with control group, in order to better understand the intra- and inter-population variability exhibited by patients.

Before further discussions, some aspects of this study based on facial photographs and geometric morphometric analysis must be addressed. On one hand, the use of photographs to analyze facial dysmorphology can be considered a disadvantage when compared with the analysis of 3D facial modeling, given that the latter has shown high discriminatory power in the classification of syndromes [46–48]. Furthermore, facial photographs have been reported as unreliable method when specialists examine the diagnosis of 22q11.2DS [49]. However, its analysis employs cheaper equipment and provides data that are easier to handle than 3D data, and recently, this kind of analysis has been shown to provide a promising raw material for different software packages for automatic identification of syndromes [50, 51]. On the other hand, given the diagnosis difficulties in our population, our analyses and discussions are based on a small sample size. We are aware that this study should be replicated with a bigger sample size. Considering the scarcity of clinical descriptions of Latin American populations [19], we considered the present study a first approach to the analysis of this facial dysmorphology in the Mexican population.

Facial dysmorphology has been analyzed using different approaches and methods. However, the scarcity of quantitative studies focused on the facial features of this syndrome makes the results of previous studies are not directly comparable with those obtained in the present study. Therefore, our discussions only include tendencies and general comparisons of the results, combining soft- and hard tissue studies, since it has been reported that the former is reliable proxy of the latter [52].

### Overall facial dissimilarities

It has been stated that the expression of facial characteristics in the context of 22q11.2DS may vary depending on the ethnic group [19]. For example, African and Afro-American patients have shown subtle facial dysmorphism [17, 18], with auricular abnormalities reported as more frequent than other features [53], whereas the phenotypes of Chinese and Chilean patients have been described as similar to that of Caucasian patients [54]. However, a study in European patients has observed some differences from the typical Caucasian phenotype, where patients exhibit downslanted palpebral fissures [27]. Likewise, a long face has not been associated with Japanese patients [55], although this feature has been frequently observed in the syndrome. Our results obtained in Mexican population show that (Fig. 4a) there are differences between the mean shape of patients and controls, differences consistent with the characteristics reported as typical for Caucasian population [56]. That is, patients have longer faces than controls, short and upslanting palpebral fissures, hypertelorism, shorter philtrum, downward labial commissures and short ears. Nevertheless, the nasal differences are not as marked as the other facial differences in this overall comparison, maybe due to the fact that the characteristic nose previously reported includes lateral variation (e.g. a bulbous nasal tip) which was not analyzed in this study; another likely factor is that the nasal trait that discriminates between groups in this study (i.e. hypoplasia of the nasal wings) is only observed in the older age group (Fig. 4d).

Also, facial size has been reported to be an important criterion included in the definitions and descriptions of different syndromes [57]. However, to the best of our knowledge, there are few studies that consider the analysis of facial size in 22q11.2DS and its impact on facial shape. For example, in a dense surface analysis performed in individuals pertaining to a similar age group to that of the present study, a slight increase in overall facial size was found in patients relative to controls [48]. Similarly, in the study by Sinderberry *et al*. [58], the facial size is one of the traits that statistically differentiate the two subtypes related to mental health problems in individuals with 22q11.2DS. Our results showed that patients have smaller facial size than controls when all the ontogenetic stages were taken into account, and also within each age group (see below).

In addition to explore the mean facial phenotype and size related with 22q11.2DS, we present a detailed description of the morphological variation of Mexican patients. We found that the patterns of facial covariation between patients and controls are not the main source (i.e. PC1) of variation within our sample (Fig. 2). Prasad *et al*. [48] reported a similar finding using 3D facial surfaces of affected and unaffected siblings; nonetheless, they found significant distinction between patients and controls in the second PC. Altogether, these results could support claims on the difficulty of recognizing the typical facial morphology of patients based on qualitative inspection only [14], and contrast with the facial variability observed for other syndromes. For example, Goodwin *et al*., studied the facial dysmorphology related to hypohidrotic ectodermal dysplasia using 3D imaging, and found that patients and controls differed along the first PC [59].

The present study also confirms the range of morphological variation that has been previously reported [5, 11, 14, 15, 27, 46, 56]. The facial features that showed greater morphological variation in our patients included the mouth, which was observed to be small and arc-shaped, with thinner lower lip compared to the upper lip in some patients, while in others it was found to be wide with the thinning of both lips. In addition, other variable features were observed, for instance, the palpebral fissures, which were upslanting and oblique or normal, and the implantation of the ear, which was low or normal. Finally, the facial thirds showed variability in different proportions.

These results, along with the ontogenetic-related changes would benefit the diagnosis of 22q11.2DS since it has been reported as more likely when a systematic evaluation that considers the presence of three or more of these characteristic features is performed [14]. Moreover, given that these facial traits represent the most descriptive characteristics for this group of individuals, they could be used for clinical training [32, 60]. Finally, the description of the facial phenotypic variation in patients may also be useful for the identification of greater number of individuals, thereby improving the comprehensiveness of their medical care and genetic counseling of the families.

### Ontogenetic exploration of facial dysmorphology

We explored whether part of the morphological differences found between patients and controls are related to ontogenetic processes, i.e. development, growth, and allometry. According to previous studies, facial appearance in 22q11.2DS varies among ontogenetic stages. For example, some studies have mentioned that adults have more subtle manifestations [25–27], while others have reported that newborns and patients in early childhood are more difficult to diagnose [61–63]. Our results showed a pattern of morphological differentiation between patients and controls that begin at G1, which supports the existence of prenatal or early postnatal morphological differences in our sample. Features like reduced philtrum, smaller ears and the inverted v-shaped mouth could be useful to diagnosis in all ontogenetic stages (Fig. 4, Table 3). In addition, small mouth and eyes, nasal hypoplasia, and low-set position of the ears could be included as discriminative traits at G3 (Fig. 4d).

In the same manner, we found that facial size differences have an age-specific pattern that is more pronounced at G3, where patients are 91.4% smaller than controls at such stage. This result is similar to previous reports of growth retardation associated to 22q11.2DS [34, 35, 64] related to height, weight [35] and head circumference [34] of Caucasian patients.

Growth disruption in 22q11.2DS individuals have been associated with environmental perturbations such as nutritional disorders [64] or hormonal deficiencies [13, 65]. Studies using mice as model of craniofacial growth development have confirmed that there is a differential response of the craniofacial components to such perturbations, with the viscerocranium being the most affected structure compared to the neuro- or basicranium. Gonzalez *et al*., [66] showed that in the case of postnatal malnutrition exposure, the effect of growth reduction is related to the time and magnitude of the perturbation and to the rates and relative timing of growth of the structure being affected. They found that the face component experienced larger size reduction when the exposure was late in ontogeny or was maintained throughout development and smaller reduction when the malnutrition occurred only in early ontogeny. In contrast, Gonzalez *et al*., found in mice that is deficient in growth hormone a catch-up growth pattern in which facial morphology was affected independently of the timing of treatment [67].

Along with size deficiency due to environmental disturbances, the underlying genetic basis of this syndrome could also be affecting the facial size and shape variation in patients, especially, genes critical for CHD. For example, one gene identified as crucial for cardiovascular morphogenesis in 22q11.2DS is *TBX1*, which encodes a T-box transcription factor that may disrupt the Shh or Fgf signaling pathways [68]. In avian models, this disruption has been shown to contribute to the variation of adult mid-facial growth, shape and size (e.g. brain-face covariation). Furthermore, *Tbx1* haploinsufficiency in mice produces embryos that exhibit facial dysmorphism typically associated with 22q11.2DS and that appeared smaller than the littermates [69]. In humans, a recent study of Israeli patients has indirectly supported the genetic effect on the ontogenetic disruption by finding no correlation between growth delays and palate anomalies or recurrent infections, and significant association with CHD [36].

It remains to be determined if there is a relationship between catch-up growth in the facial morphology of 22q11.2DS individuals and nutritional disturbance, hormone deficiency or genetic variability. In the first case, since the amount of exposure to poor diet could have different outcomes on the facial phenotype, and feeding difficulties are frequently observed in early ontogeny (i.e. infants and children) given velopharyngeal insufficiency and other gastrointestinal complications [4, 70]. In the second case, because, although it has been shown that there is a low prevalence of growth hormone deficiency in 22q11.2DS individuals, those patients with this endocrine disorder treated with growth hormone show growth improvement [36], even when an adequate dietary intake showed no results [71]. In the last case, given that CHD is commonly observed in patients and a recent study shows promising results that suggest that the embryonic treatment using vitamin B12 ameliorates *Tbx1* gene haploinsufficiency in mice [72]. This latter result could be relevant for the population analyzed in the present study, since CHD was highly prevalent (91.9%) in patients, as it has been reported in a previous study of Mexican patients [73].

The overall growth disruption observed in the present study could be explained altogether by environmental and genetic factors associated with the syndrome. Equally important, our results showed that whatever the cause, such size variation influences differently the shape of patients and controls, and that these allometric differences may help to explain the phenotypic facial variability of the syndrome. In this regard, we found that patients and controls share similar developmental and allometric trends, but they differ in allometric scaling (Fig. 6b). Our study showed that, in controls, the facial shape changes related to the developmental and allometric processes share similarities (Fig. 7a and b). For instance, in both cases (i.e. young/old and small/large comparisons) we observed a less protrusive position of the ears and an enlargement of the middle and lower third of the face outline that includes changes in the nose and lips. On the contrary, although patients exhibited a similar change in the facial outline in both processes, there were differences in the midline structures, that is, eyes, nose, and lips (Fig. 7c and d).

Taken together, these results suggest that facial dysmorphology within our population is not further accentuated by alterations of the amount of shape change occurring over time (development) or by modifications of the shape and size relationship (allometry). Nevertheless, it seems to be affected by truncation of the ontogenetic trajectory due to the underlying growth delay experienced by patients (Fig. 5). These same mechanisms have been suggested as possible explanations of the contrasting adult morphologies between populations; for instance, Inuit and Khoisan [31], or Caucasians and African-Americans [30]. At the same time, it has been shown [19, 20] that 22q11.2DS individuals of the latter populations differ in the degree of severity of the facial phenotype. Therefore, it remains to be explored if some amount of inter-population variability exhibited by these 22q11DS patients is related to the already present developmental differences.

In developing countries, where only limited resources are available to conduct molecular biology-based tests (e.g. Multiplex Ligation-dependent Probe Amplification, Fluorescence in situ hybridization or CGH microarray) in disorders like 22q11.2DS, it is of utmost importance to establish the robustness of patients’ clinical features, like facial appearance, as valid elements to reach a diagnosis. This is especially true when canonical diagnostic criteria have been developed in populations with different ancestry.

## Conclusion

The main contribution of our study lies in that it constitutes a first approach to the quantitative assessment of facial variability across 22q11.2DS patients and its relationship to postnatal ontogeny compared with non-affected controls in our population. We found that Mexican patients exhibited typical traits that have been reported for the Caucasian population, and that those traits differ significantly from those of controls on average and at each ontogenetic stage. In addition, we found that the developmental and allometric trajectories of patients and controls were similar, but that they differ in facial growth patterns.

The clinical implications of our findings are that even though patients exhibit large variability of facial characteristics, features like the downward labial commissures, the reduced philtrum and the smaller ears could be useful to diagnosis in all ontogenetic stages, while upslanting palpebral fissures, a longer midface and hypertelorism could be included in the diagnosis from childhood, and traits like nasal hypoplasia and low-set position of the ears until adolescence. In addition, given that postnatal ontogeny could influence the dysmorphology and its variability through size-related changes, it may be possible to find less marked typical facial traits in patients with no growth restrictions; however, more research is needed in this regard.

Our findings could be valuable in clinical training and, given the shared ancestry that our population possesses with that of most Latin-American countries, could be applied to a significant fraction of the world’s population. Additionally, we have a better understanding of the impact that postnatal ontogeny may have on facial variability of patients with 22q11.2DS.

## Data Availability

Not applicable.

## Abbreviations

22q11.2DS: 22q11.2 deletion syndrome
2D: Second dimension
3D: Third dimension
CHD: Congenital heart disease
FISH: Fluorescent in situ hybridization
GM: Geometric morphometric
PCA: Principal component analysis
PCs: Principal components
VCFS: Velocardiofacial syndrome
